# Overexpression of the C4 protein of tomato yellow leaf curl Sardinia virus increases tomato resistance to powdery mildew

**DOI:** 10.3389/fpls.2023.1163315

**Published:** 2023-03-31

**Authors:** Chiara D’Errico, Marco Forgia, Marco Pisani, Stefano Pavan, Emanuela Noris, Slavica Matić

**Affiliations:** ^1^ Institute for Sustainable Plant Protection, National Research Council, Turin, Italy; ^2^ European Laboratory for Non-Linear Spectroscopy, Sesto Fiorentino, Italy; ^3^ Istituto Nazionale di Ricerca Metrologica, Applied Metrology and Engineering Division, Torino, Italy; ^4^ Department of Soil, Plant and Food Sciences, University of Bari “Aldo Moro, Bari, Italy

**Keywords:** tomato, geminivirus, C4 protein, *Oidium neolycopersici*, biotic stress, gene expression, phytohormones, transgenic plant

## Abstract

Powdery mildew (PM) is one of the most important diseases of greenhouse and field-grown tomatoes. Viruses can intervene beneficially on plant performance in coping with biotic and abiotic stresses. Tomato yellow leaf curl Sardinia virus (TYLCSV) has been reported recently to induce tolerance against drought stress in tomato, and its C4 protein acts as the main causal factor of tolerance. However, its role in response to biotic stresses is still unknown. In this study, transgenic tomato plants carrying the TYLCSV C4 protein were exposed to biotic stress following the inoculation with *Oidium neolycopersici*, the causal agent of tomato PM. Phytopathological, anatomic, molecular, and physiological parameters were evaluated in this plant pathosystem. Heterologous TYLCSV C4 expression increased the tolerance of transgenic tomato plants to PM, not only reducing symptom occurrence, but also counteracting conidia adhesion and secondary hyphae elongation. Pathogenesis-related gene expression and salicylic acid production were found to be higher in tomato transgenic plants able to cope with PM compared to infected wild-type tomato plants. Our study contributes to unraveling the mechanism leading to PM tolerance in TYLCSV C4-expressing tomato plants. In a larger context, the findings of TYLCSV C4 as a novel PM defense inducer could have important implications in deepening the mechanisms regulating the management of this kind of protein to both biotic and abiotic stresses.

## 1 Introduction

Tomato (*Solanum lycopersicum* L.) is one of the most agronomically important food crops, with a total of 186 million tons globally produced in 2020 on more than 5 million hectares, out of which approximately 0.1 million hectares are cultivated in Italy (FAOSTAT; https://www.fao.org/faostat/en/#data/QCL). Although the tomato market is continuously growing, a large number of different pathogens affect this crop, inducing significant yield losses (Panno et al., 2021). Powdery mildew (PM) is a widespread disease affecting more than 10,000 different botanical species. It is caused by several ascomycete fungi (order *Erysiphales*), ectoparasites that act as obligate biotrophic organisms (Takamatsu, 2004). On tomatoes, PM is caused frequently by *Oidium neolycopersici* L. Kiss (*On*), which is considered a worldwide emerging pathogen that induces white powdery lesions on the upper surface of the leaves. White powdery colonies may also develop on lower leaf surfaces during late disease stages and, in case of severe epidemics, petioles, stems, and sepals, but not fruits can be attacked (Jones et al., 2001; Jacob et al., 2008). *On* causes significant yield losses both in the greenhouse and in the open field, favoring temperate and humid climates. These yield losses might be exacerbated by events related to the ongoing climate change, particularly increased humidity following heavy rainfalls (Jacob et al., 2008).

New global pathogen containment strategies are being developed to foster the transition towards sustainable farming practices and sustainable lifestyles, based on reduced pesticide use and on the selection of low-risk active substances. Innovative strategies are becoming fundamental as many previously registered pesticides are being withdrawn from commercialization, due to the development of resistance in the target pathogen or because of toxicity concerns. One of these strategies implies the use of biological control agents that may protect the plant from pathogen attack in different ways (e.g., competition for nutrients and space), complying with a sustainable disease management approach. In nature, combinations of virus and other pathogen infections cause the activation of overlapping or synergistic molecular and metabolic mechanisms, which can change the outcome of the disease epidemics (Lehtonen et al., 2006; Syller and Grupa, 2016; Köhl et al., 2019). Furthermore, there are reports of virus infection reducing the incidence of other pathogens in the same plants, including PM fungi, such as the cases of barley yellow dwarf virus, potato virus Y, and zucchini yellow mosaic virus (ZYMV) active against *Blumeria graminis* f. sp. *hordei*, *Erysiphe cichoracearum*, and *Podosphaera* ssp., respectively (Potter and Jones, 1981; Marte et al., 1992; Harth et al., 2018). However, these reports are mainly focused on the phytopathological aspects of the plants and do not thoroughly describe the key molecular and phytohormonal aspects involved in this kind of defense reaction.

Pathogen recognition activates signaling cascades resulting in conserved defense responses that can also act against other co-infecting viral and nonviral agents or towards subsequent parasite attacks (Jones and Dangl, 2006; Teixeira et al., 2021). Signaling cascades mainly operate through the recruitment of phytohormones, mostly salicylic acid (SA) and jasmonic acid (JA), which are key actors of plant defense strategies. Specifically, SA is considered the primary phytohormone that mediates resistance against biotrophic pathogens (An and Mou, 2011), while JA is primarily responsible for regulating defense responses against necrotrophic pathogens and herbivores (Wasternack, 2007). SA-dependent disease resistance was reported in the case of the PM-resistant 4 (*pmr4*) *Arabidopsis* mutant line (Nishimura et al., 2003) and in the PM partially resistant tomato line carrying the *Ol-1* resistance gene when subjected to single (PM) or combined (PM and drought) stress (Sunarti et al., 2022). In the last decade, the biochemical processes underlying the infection and the resistance responses of tomato plants to *On* are being investigated, including the role not only of hormones (Achuo et al., 2004; Li et al., 2012), reactive oxygen species (ROS) (Mlícková et al., 2004; Tománková et al., 2006), and reactive nitrogen species (Piterková et al., 2009; Piterková et al., 2013), but also of elicitors (oligandrin, BABA) (Satková et al., 2017), whose effect can be related either to effector-triggered immunity (ETI) or to pathogen-associated molecular pattern (PAMP)-triggered immunity (PTI).

A crosstalk between responses to different stresses exists and plants carrying genes from certain pathogens were shown to display resistance towards other pathogens, such as bacterium *vs*. virus and yeast *vs.* virus (Cao et al., 2013; Zhirnov et al., 2016; Yang et al., 2019). Given that plant disease management is continuously evolving and that the presence of viruses in plants can also have beneficial outcomes (Gorovits et al., 2022), we investigated if the presence of viruses or the expression of viral proteins in plants may be beneficial against other pathogen infections.

Geminiviruses are circular single-stranded DNA viruses belonging to one of the largest families of plant viruses (*Geminiviridae*), infecting an extremely broad number of hosts (Fiallo-Olivé et al., 2021). Several species from this family have the potential to trigger and overcome the complex antiviral immune response of the plant, which include RNA interference (RNAi), ETI, and PTI (Teixeira et al., 2021).

The C4**/**AC4 proteins of geminiviruses are the less conserved proteins within this family, display a broad diversity of functions during viral infection, and have been implicated in several functions (Medina-Puche et al., 2021), such as virus movement (Jupin et al., 1994; Rojas et al., 2001), suppression of RNA silencing (Amin et al., 2011; Luna et al., 2012; Ismayil et al., 2018; Rosas-Diaz et al., 2018), symptom induction (Rigden et al., 1994), promotion of hypersensitive response (Mei et al., 2020), and hyperplasia (Jing et al., 2019). Recently, we reported that the C4 protein of tomato yellow leaf curl Sardinia virus (TYLCSV) empowers drought stress tolerance in transgenic tomato plants overexpressing it (Pagliarani et al., 2022), similarly to the C4 protein of a related begomovirus, i.e., tomato yellow leaf curl virus (TYLCV) (Corrales-Gutierrez et al., 2020; Gorovits et al., 2022; Mishra et al., 2022). These TYLCSV-C4 overexpressing lines C4-151, -153, and -156 exhibit varying degrees of phenotypic alterations, including deformed leaves with a curly and crispy morphology, and a reduced size showed considerable level of resistance to drought compared to wild-type (WT) individuals. To date, the role of the C4 protein in tackling biotic stress responses remains elusive.

In this work, we explored whether the transgenic expression of the TYLCSV C4 protein, besides being beneficial for the plant response to abiotic stresses, might also have a role in the response to biotic stresses. For this purpose, we inoculated TYLCSV C4-expressing tomato plants with the biotrophic pathogen *On*, measuring the progress of the PM disease in both WT and transgenic plants and exploring molecular, anatomic, physiological, and phytohormonal responses. The expression of the TYLCSV C4 protein induced tolerance to PM disease in tomato plants, mainly through the modulation of stress marker genes and the biosynthesis of the SA phytohormone, providing novel insights into the molecular and phytohormone mechanisms tackled by this multifunctional viral component.

## 2 Materials and methods

### 2.1 Fungal maintenance

The isolate MB1 of the pathogenic fungus *On* originating from commercial tomato plants was maintained on tomato WT plants (cv. Moneymaker) in a greenhouse compartment at 23°C (day) and 19°C (night) with 70% relative humidity. ITS sequence was amplified through PCR using ITS4 and ITS5 primers (White et al., 1990) on DNA extracted from *On*. The obtained PCR amplicon (594 bp) was sequenced through the Sanger method (Biofab s.r.l., Rome, Italy) to identify the PM species as *On* (**Supplementary File 1**).

### 2.2 Experimental conditions

To evaluate if TYLCSV C4 expression has an impact on the infection by *On*, tomato plants of lines C4-151, C4-153, and C4-156 overexpressing the viral *C4* gene under the control of the constitutive 35S promoter (Pagliarani et al., 2022) were artificially inoculated with *On*, using WT tomato plants (cv. Moneymaker) as controls. As all transgenic lines exhibited reduced PM symptomatology, further systematic experiments were conducted on the C4-151 line whose plants exhibit a homogeneous morphology which was described in detail by Pagliarani et al. (2022).

In detail, T3 plants of the C4-151 line, together with WT control plants, were grown in a glasshouse, under partially controlled climatic conditions, as described above. Each plant was grown in a 6-L pot filled with a substrate composed of sandy-loam soil/expanded clay/peat mixture (3:2:4 by volume). Two months after sowing, nine plants of each genotype were inoculated on two primary leaflets of three randomly selected leaves, using 10 µl of an *On* suspension (5 × 10^4^ spores ml^−1^) prepared by washing conidial spores from leaves of heavily infected (sporulation stage) plants. After inoculation, plants were grown for another 20 days, daily monitoring the PM development. The experiment was repeated twice during the same season and the results shown in the manuscript have been mediated between the two repetitions.

### 2.3 Plant and fungal performanceе

The presence of *On* symptoms was assessed by visual inspection at 8, 13, and 15 days post-inoculation (dpi), according to Bai et al. (2008). Plants were photographed with a Casio Exilim EX-Z85 digital camera and images were analyzed with a dedicated Wolfram Mathematica script (Long Hanborough, UK). Depending on the stage of symptoms developed, the area of infection on individual leaves was quantified either considering the PM colonized region (white color) or the necrotic one (brown color), based on changes deduced from the RGB (red, green, and blue) values. The disease index (DI) was assessed at the same time points and on the same leaflets, using a scale from 0 to 4, according to Bai et al. (2008) and Patil et al (Patil and Bodhe, 2011), as follows: 0, apparently not infected; 1, 0%–25% leaf area infected; 2, 25%–50% leaf area infected; 3, 50%–75% leaf area infected; 4 >75% leaf area infected.

### 2.4 Chlorophyll content index

The chlorophyll content index (CCI) was determined on the second, third, and fourth fully developed leaves counting from the plant apex of three plants (nine leaflets in total), using a portable chlorophyll meter (SPAD 502, CCM-200; Opti-Sciences, Hudson, NH, USA).

### 2.5 Analysis of adhesion and elongation of germ tubes and secondary hyphae of *On* conidia

Conidia density and elongation of germ tubes and secondary hyphae of *On* were determined with scanning electron microscope (SEM) (TM3000, Hitachi High-Technologies Corp., Tokyo, Japan) images (120× magnification). For each group, three plants were observed, counting conidia of two leaves each.

### 2.6 Analysis of hormone content

Hormone content was quantified as reported in Pagliarani et al. (2022) and Nerva et al. (2022). Following freeze drying, 40 mg of tissue was homogenized and extracted in an ultrasonic bath for 1 h with 1 ml of a mixture of methanol:water (1:1, v/v), acidified with 0.1% formic acid. After centrifugation (15,000 rpm, 10 min, 4°C), the supernatant was used to quantify abscisic acid (ABA), indole-3-acetic acid (IAA), and SA, adopting the external standard technique, with calibration curves made with original analytical standards (all from Sigma Aldrich, purity ≥98.5% for ABA and ≥99% for IAA and SA). The HPLC apparatus (Agilent 1220 Infinity LC system model G4290B, Agilent, Waldbronn, Germany) was equipped with a gradient pump, an autosampler, and a column oven set at 30°C. A 170 Diode Array Detector (Gilson, Middleton, WI, USA) set at 265 nm was employed, using a Nucleodur C18 analytical column (5 μm length: 250 mm, ID: 4.6 mm, Macherey-Nagel GmbH & Co. KG, Düren, Germany). The mobile phases were water acidified with 0.1% formic acid (A) and acetonitrile (B), at a flow rate of 0.600 ml min^−1^ in gradient mode, 0–6 min: from 10% to 30% of B, 6–16 min: from 30% to 100% B, 16–21 min: 100% B. Twenty microliters per sample was injected, testing all biological replicates (*n =* 3).

### 2.7 Total RNA isolation and quantitative real-time PCR

Total RNA was extracted from 100 mg of leaf sample using Trizol (Thermo Fisher Scientific, Waltham, MA, USA) and treated with TURBO DNase (Ambion, Waltham, MA, USA) to eliminate DNA contamination. cDNA was synthesized from 500 ng of total RNA with the high-capacity cDNA reverse transcription kit (Applied Biosystems, Waltham, MA, USA), incubating samples at 25°C for 10 min, then at 37°C for 2 h , and finally at 85°C for 5 min. The qRT-PCR analysis was carried out in a CFX96 Real-Time PCR Detection system (Bio-Rad, Hercules, CA, USA). The mixture consisted of 1 µl of cDNA, 1× iTaq Universal SYBR Green Supermix (Bio-Rad), and 0.25 µM of each primer. Thermal cycling conditions included an initial denaturation at 95°C for 10 min, followed by 40 cycles at 95°C for 15 s and 60°C for 1 min. Specific annealing of primers was checked on dissociation kinetics performed at the end of each qRT-PCR run. The expression level of each tomato target transcript was normalized to the geometric mean of the elongation factor (*SlEF*) and ubiquitin (*SlUBI*) transcripts, used as endogenous controls, according to the 2^−ΔΔCT^ method (Livak and Schmittgen, 2001). Three biological replicates, each with three technical repetitions, were tested for each sample for every condition. Gene-specific primers used in the qRT-PCR experiments are listed in **Table 1**.

**Table 1:** List of gene-specific primers used for qRT-PCR.

Primer name	Sequence	Ref.
PR1b1_F_	GCACTAAACCTAAAGAAA	(Fiocchetti et al., 2006)
PR1b1_R_	TAGTTTTGTGCTCGGGATGC	
SR1a4_F_	GTGTCCGAGAGGCCAGACTA	(Kissoudis et al., 2016)
SR1a4_R_	CATTGTTGCAACGAGCCCGA	
SlPR2_F_	TCCAGGTAGAGACAGTGGTAAA	(Li T. et al., 2019)
SlPR2_R_	CCTAAATATGTCGCGGTTGAGA	
SlPR5_F_	GGCCCATGTGGTCCTACAAA	(Li T. et al., 2019)
SlPR5_R_	GGCAACATAGTTTAGCAGACCG	
SlPOD_F_	TTGGAGTGTCTCGTTGCTCA	(Li T. et al., 2019)
SlPOD_R_	TTCACCAGCACTCCCTGTCT	
SlNPR1_F_	GGGAAAGATAGCAGCACG	(El Oirdi et al., 2011)
SlNPR1_R_	GTCCACACAAACACACACATC	
SlNPR3_F_	CTGAGGTTCTTGGACTGGGT	This study
SlNPR3_R_	GCCTAGTCAGCCTCCTACAG	

### 2.8 Statistical analyses

**Figure 1:**
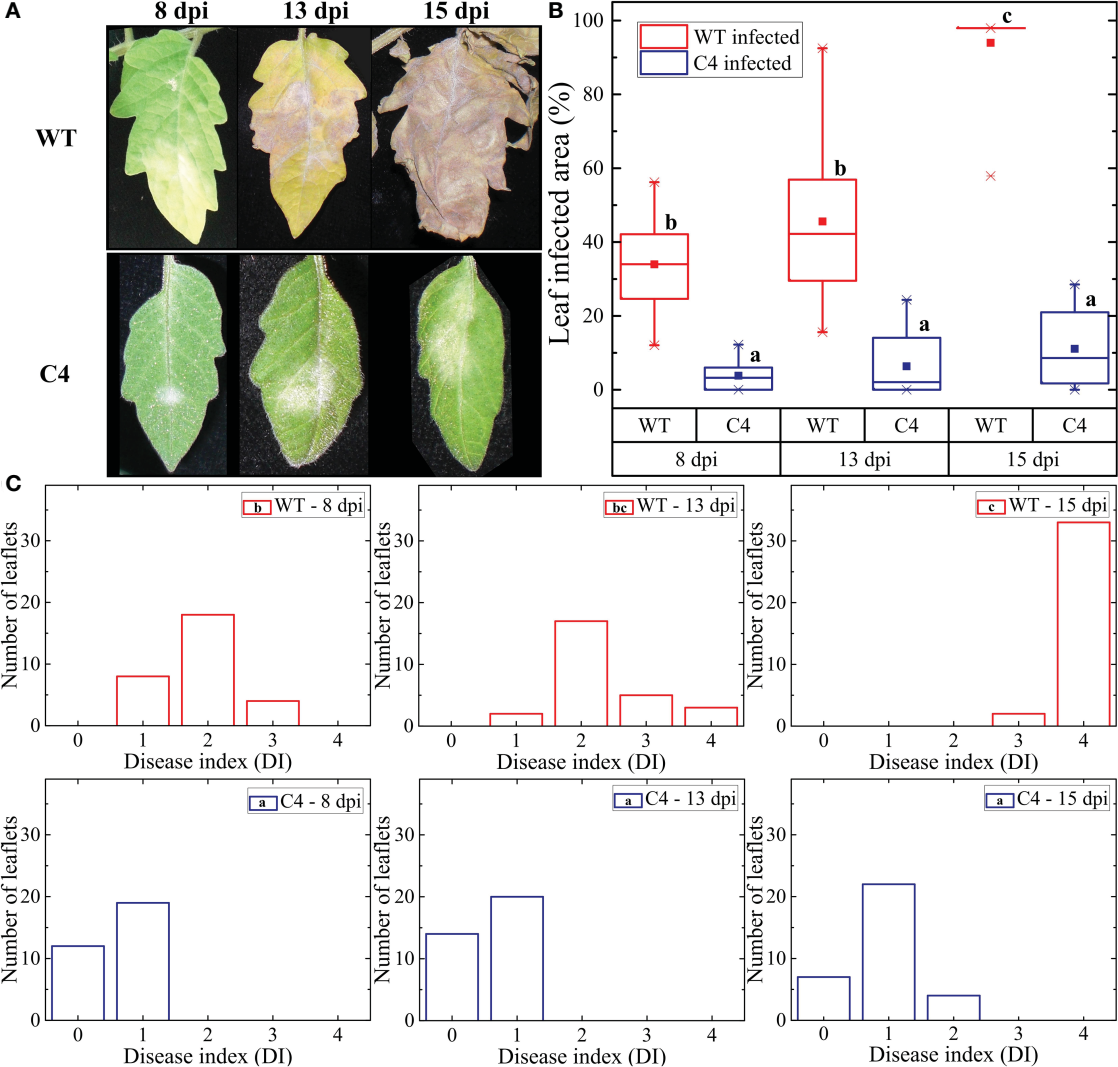
Effect of *Oidium neolycopersici* (*On*) infection on tomato Moneymaker wild-type (WT) and transgenic TYLCSV C4-expressing plants. **(A)** Leaflets of *On*-inoculated WT and C4 plants photographed at 8, 13, and 15 days post-inoculation (dpi). **(B)** Percentage of diseased area calculated using a dedicated Wolfram Mathematica script based on changes in RGB (red, green, and blue) values. For each group, the box plot chart refers to 36 leaflets from six biological replicates and reports the mean (filled square), the median (solid horizontal line), the first and the third quartile (box), 1.5 * interquartile range (IQR) below the first quartile and above the third quartile (whiskers), and the 1st and 99th percentiles (x symbols). Kruskal–Wallis test was carried out with multiple comparisons between groups and a statistically significant difference was accepted at the 1% probability level. Groups with the same letter do not have significant differences according to the *post-hoc* Dunn’s test at the 5% probability level. **(C)** Values of disease index (DI) calculated using image processing of leaflets based on the DI scale described in Patil et al (Patil and Bodhe, 2011). Statistical significance between groups was calculated as described in **(B)**.

**Figure 2:**
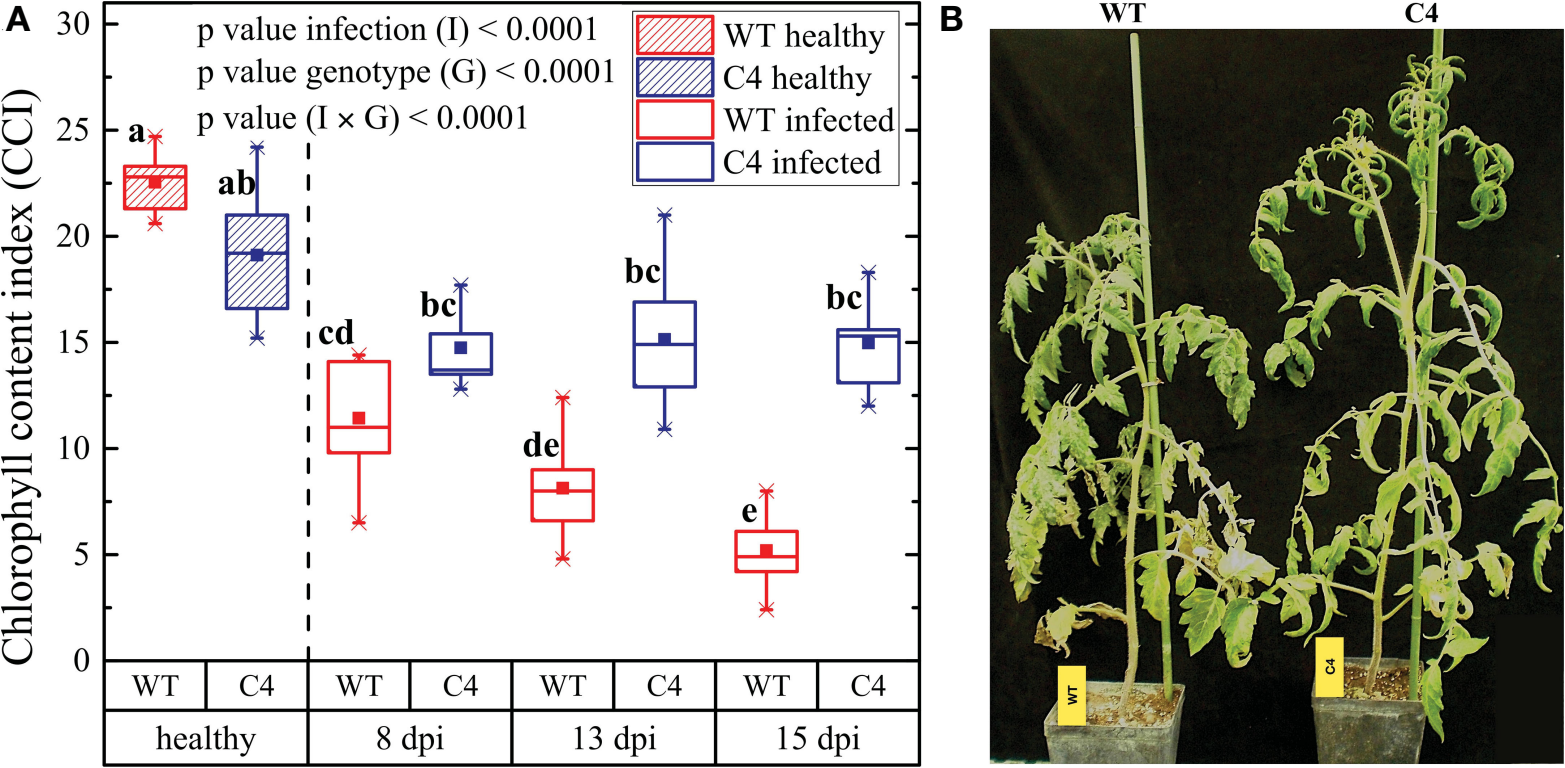
Impact of *Oidium neolycopersici* (*On*) infection on wild-type (WT) and TYLCSV C4-expressing tomato plants. **(A)** Chlorophyll content index (CCI) of healthy and *On*-infected WT and C4-expressing tomato plants at 8, 13, and 15 dpi. For each group, the box plot chart refers to nine leaflets from three biological replicates and reports the mean (filled square), the median (solid horizontal line), the first and the third quartile (box), 1.5 * interquartile ranges (IQR) below the first quartile and above the third quartile (whiskers), and the 1^st^ and 99^th^ percentiles. The significance of genotype (G), infection (I), and genotype × infection (G × I) was assessed by two-way ANOVA. Groups with the same letter do not have significant differences according to the Tukey test at the 1% probability level. **(B)** Photographs of representative examples of WT and C4 *On-*infected plants at 15 dpi.

**Figure 3:**
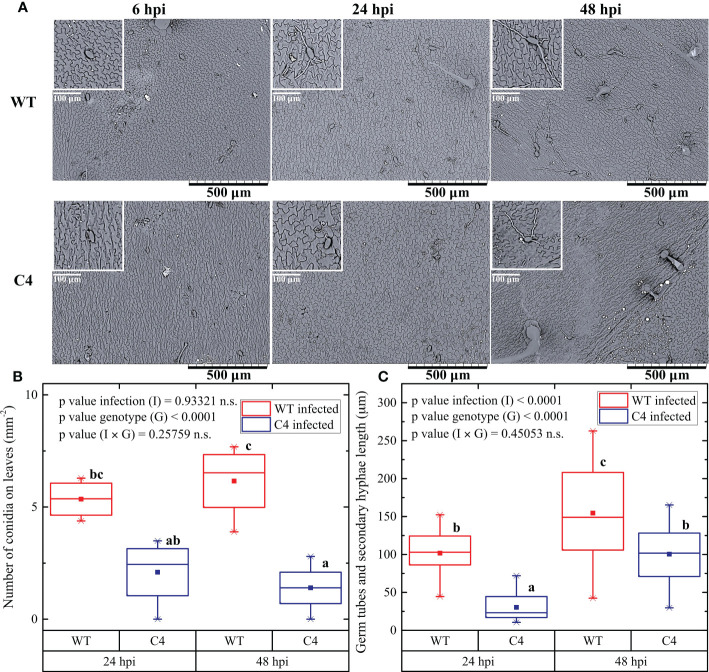
Effect of TYLCSV C4-expression on the *Oidium neolycopersici* (*On*) conidia adhesion, the emergence of germ tubes, and elongation of secondary hyphae on the leaf surface. **(A)** Scanning electron micrographs of the upper surface of WT and C4 tomato leaves at 6, 24, and 48 hours post-inoculation (hpi) of *On*. Scale bar is as indicated in each image. **(B)** Number of *On* conidia on leaves after 24 and 48 hpi. **(C)** Length of germination tubes and secondary hyphae after 24 and 48 hpi. For each group, three plants were considered, counting conidia of two leaves each. For each group, the mean (filled square), the median (solid horizontal line), the first and the third quartile (box), 1.5 * interquartile ranges (IQR) below the first quartile and above the third quartile (whiskers), and the 1^st^ and 99^th^ percentiles. The significance of genotype (G), infection (I), and genotype × infection (G × I) was assessed by two-way ANOVA. Lowercase letters above bars are reported when G × I interaction or infection (I) main effects are statistically significant as attested by the Tukey test at the 1% probability level.

**Figure 4:**
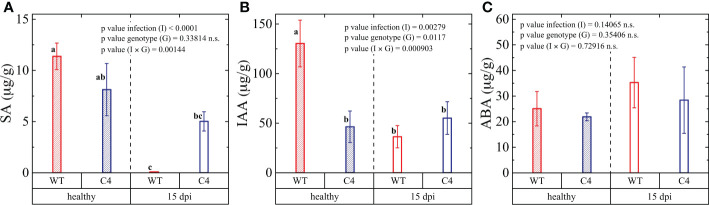
Hormone regulation in wild-type (WT) and TYLCSV C4 tomato plants infected by *Oidium neolycopersici*. The content of **(A)** salicylic acid (SA), **(B)** indole-3-acetic acid (IAA), and **(C)** abscisic acid (ABA) evaluated 15 days post-inoculation (dpi). The pool of nine leaflets from three biological and three technical replicates for each group was presented. The significance of genotype (G), infection (I), and genotype × infection (G × I) was assessed by two-way ANOVA. Lowercase letters above bars are reported when G × I interaction and infection (I) main effects are statistically significant as attested by Tukey test at the 1% probability level. Error bars represent standard deviation (SD).

**Figure 5:**
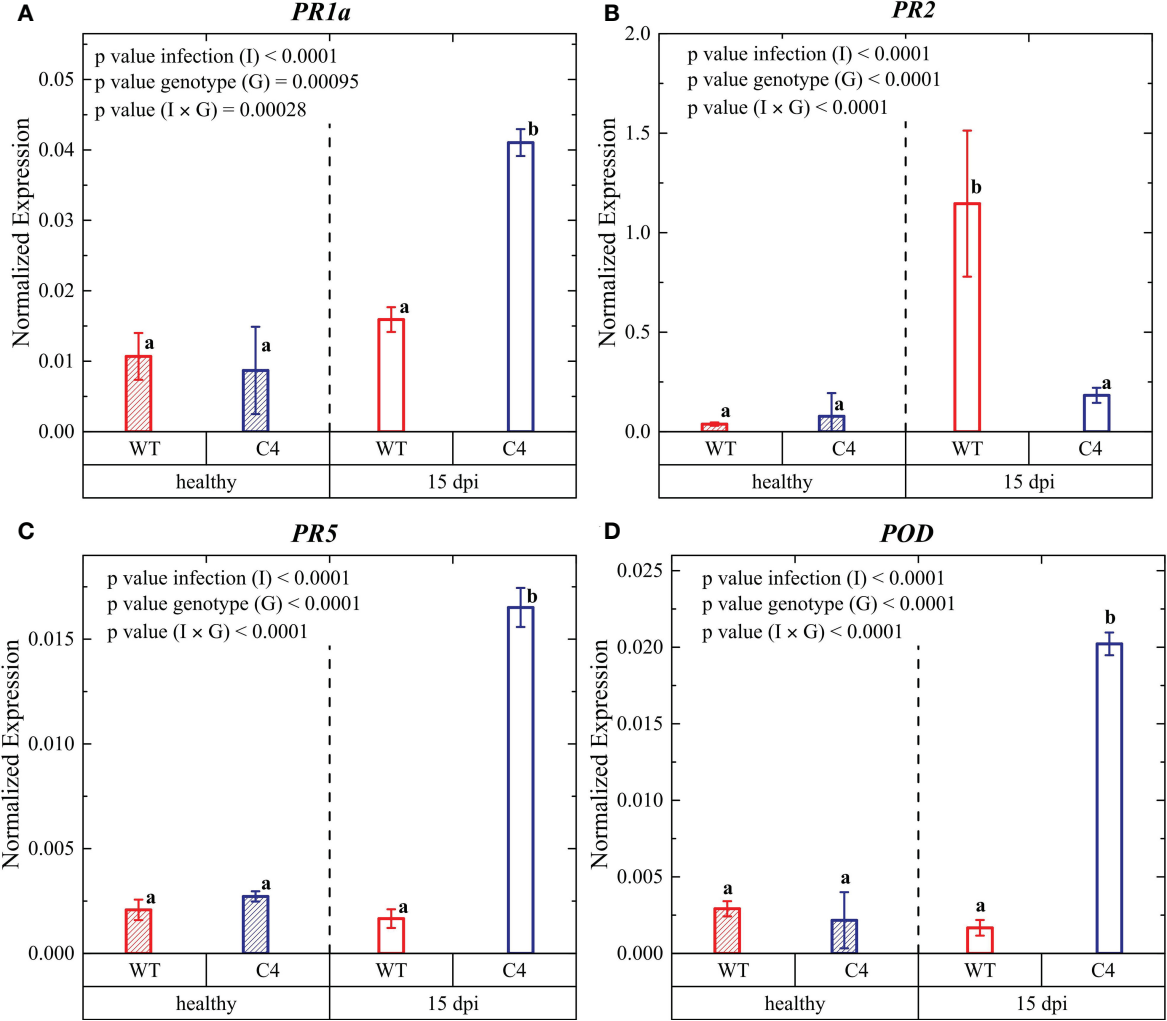
Expression profiles of key late defense genes involved in biotic stress signaling pathways in leaves from WT and TYLCSV C4 tomato plants infected with *Oidium neolycopersici* (*On*) at 15 days post-inoculation (dpi): *PR1a* **(A)**, *PR2* **(B)**, *PR5* **(C)**, and *POD* **(D)**. Ubiquitin and elongation factor 1α genes were both used as endogenous housekeeping controls for the normalization of transcript levels. Significance of genotype (G), infection treatment (I), and infection × genotype (I × G) interaction was assessed by two-way ANOVA. Groups with the same letter do not have significant differences according to Tukey test at the 1% probability level. Error bars represent standard deviation (SD). For expression analyses, three independent biological replicates with three technical replicates each were used.

**Figure 6:**
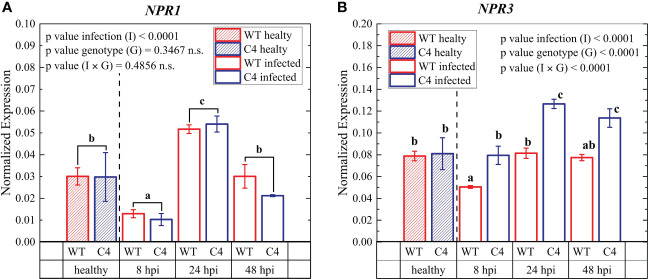
Expression profiles of key early defense genes involved in biotic stress signaling pathways in leaves from wild-type (WT) and TYLCSV C4 tomato plants infected by *Oidium neolycopersici* (*On*) at 8, 24, and 48 hours post-inoculation (hpi) *NPR1* **(A)** and *NPR3* **(B)**. Ubiquitin and elongation factor 1α genes were both used as endogenous housekeeping controls for the normalization of transcript levels. Significance of genotype (G), infection treatment (I), and infection × genotype (I × G) interaction was assessed by two-way ANOVA; n.s. = not significant. Groups with the same letter do not have significant differences according to Student’s *t*-test (a) or Tukey test (b) at the 1% probability level. Error bars represent standard deviation (SD). For expression analyses, three independent biological replicates with three technical replicates each were used.

The significance of sampling genotype (G), infection (I), and genotype × infection (G × I) interaction was performed by running a two-way analysis of variance (ANOVA), or Kruskal–Wallis test, for non-normally (**Figure 1**), or normally (**Figures 2**–**6**) distributed datasets, respectively. When results of the ANOVA test indicated that either genotype (G: “WT”, “C4”) or infection (I: healthy, infected at different time points) or their interaction (G × I) was significant, the Tukey’s honestly significant difference (HSD) *post-hoc* test (**Figures 2**–**5**, **6B**) or the Student’s *t*-test (**Figure 6A**) was used to separate the means at the 1% probability level. The *post-hoc* Dunn’s test has been used to separate means at the 5% probability level for datasets of **Figure 1**. OriginPro software (Northampton, MA, USA) was applied for statistical elaborations and graphs.

## 3 Results and discussion

### 3.1 TYLCSV C4 transgenic plants exhibit reduced symptoms of *On* infection

The morphological features of the TYLCSV C4 transgenic lines used in this work, mainly consisting of convoluted, crumpled, and downward curled leaves (**Supplementary Figure 1**), were previously described in detail (Pagliarani et al., 2022). During the fungal infection experiments, typical PM symptoms induced by *On* started to become visible on leaves of WT plants at 6 dpi. As shown in **Figure 1A**, in these plants, the initially localized lesions evolved to necrosis by 13 dpi, along with the onset of diffuse chlorosis over the rest of the leaflets. Two days later (15 dpi), the whole leaf became completely necrotic. Since 8 dpi, all (100%) leaves of artificially inoculated WT plants showed *On* lesions (**Supplementary Table 1**). In the case of plants of lines C4-151, C4-153, and C4-156 overexpressing the viral C4 gene (Pagliarani et al., 2022), PM symptoms started to become evident at approximately 8 dpi, but fungal lesions were visible only in 60%–70% of leaves of inoculated plants (**Supplementary Table 1**). At later observation times (13 and 15 dpi), the percentage of symptomatic leaves ranged from 60% to 85% and 80% to 90%, respectively (**Supplementary Table 1**); moreover, fungal lesions remained typically restricted to a portion of the leaflets, without evolving to necrosis (**Figure 1A**).

To more precisely quantify the effect of fungal infection and to be in line with our previous observation in the context of abiotic stress (Pagliarani et al., 2022), we focused on the C4-151 line, which displayed attenuated *On* symptoms. Thus, the extension of the affected leaflet area was evaluated at each of the above time points on both WT and C4 plants. In WT plants, 34% of the leaflet area was affected at 8 dpi, reaching approximately 46% and 94% at 13 and 15 dpi, respectively (**Figure 1B**). Conversely, for C4 plants, the leaflet-affected areas were approximately 4%, 6%, and 11% at 8, 13, and 15 dpi, respectively, the latter corresponding to an 83% reduction compared to WT plants (**Figure 1B**). Therefore, expression of TYLCSV C4 leads to a dramatic reduction of *On* symptoms compared to non-transgenic plants.

Further confirmations of these results were obtained measuring the incidence of the PM through a DI scale ranging from 0 to 4. Indeed, the DI of C4 plants was significantly lower than that of WT plants, at all the time points considered (**Figure 1C**). In particular, at the end of the experiment (15 dpi), 94% of leaflets of WT plants reached the highest DI level of 4, while at the same time point, the majority (67%) of leaflets of transgenic TYLCSV C4 plants reached DI 1, and none of them overcame the DI of 2, a value overall lower than that of WT plants at 8 dpi (**Figure 1C**).

Altogether, the level of *On* symptom reduction here described for TYLCSV C4-expressing tomato plants is similar to that reported for the Arabidopsis line *pmr4-1* (Nishimura et al., 2003) and the tomato *Ol-1* line (Sunarti et al., 2022).

### 3.2 *On* infection has a lower impact on the chlorophyll content in TYLCSV C4-expressing tomato plants

The infection by biotrophic organisms is an energy-consuming process, due to the activation of plant defense responses and the competition for nutrients. Therefore, during plant–pathogen interactions, the photosynthetic process can be altered, as is the case of plants infected by *On*. Here, we evaluated the CCI of WT and C4-expressing transgenic plants inoculated with *On*. The CCI of uninfected WT and C4 plants did not statistically differ, in agreement with our previous data (Pagliarani et al., 2022) supporting the assumption that C4 does not impact the photosynthetic process. Following *On* inoculation, a strong decrease in CCI occurred in WT plants along with time progression, while the CCI value remained almost unaltered in C4-expressing plants (**Figure 2A**), a result in line with their reduced *On* symptoms. In addition, as shown in **Figure 2B**, WT infected plants were significantly smaller than *On* infected C4-expressing plants (124 ± 8 cm *vs.*149 ± 15 cm), possibly as the result of reduced photosynthesis. Since healthy C4-expressing plants are smaller than WT ones (Pagliarani et al., 2022), it is possible that C4 plants are less affected by the reduction of plant growth induced by *On*.

The strong impact on CCI recorded for WT tomato plants upon PM infection is in line with the trend reported by Kubienová et al (Kubienova et al., 2013). at early stages of infection and fits also with the behavior of plants at late infection stages, as previously predicted (Prokopová et al., 2010). Interestingly, the attenuated CCI decrease here observed for TYLCSV C4 plants challenged by *On* is in line with the results reported for tomato lines resistant to PM, which were obtained following partial introgression of the PM resistance genes *Ol*, such as the *Ol-1* and *Ol-4* (Bai et al., 2005)**;** these plants were partially resistant to *On* when subjected to mild or severe drought stress (Sunarti et al., 2022), suggesting the hypothesis that C4 mimics the priming of drought stress imposition on fungal infection. Moreover, the lower impact of *On* infection on plant height observed for C4 plants compared to WT individuals is in line with the results of the *Arabidopsis* line *pmr4-1* and the tomato line *Ol-1*, displaying partial PM resistance upon fungal inoculation (Nishimura et al., 2003; Sunarti et al., 2022).

### 3.3 TYLCSV C4 influences *On* conidia adhesion and secondary hyphae elongation

To provide insights into the mechanisms responsible for the delayed and reduced infection by *On* in the transgenic TYLCSV C4-expressing plants, the initial steps of fungal infection were explored. For this, a new inoculation experiment was conducted and both the number of conidia present on leaves and the emergence of germination tubes and secondary hyphae elongation were evaluated a few hours after the inoculation, using SEM observations. At 6 hours post-inoculation (hpi), no difference in the number of conidia was detected between WT and C4-expressing leaves (**Figure 3A**). However, at later observations, the number of conidia on leaves of WT plants was significantly higher than in C4-expressing plants, i.e., 5.4 ± 0.9 and 6.2 ± 1.6 conidia per mm^2^ at 24 and 48 hpi in WT plants, respectively, *vs*. 2.1 ± 1.5 and 1.4 ± 1.1 per mm^2^ in C4 plants (**Figures 3A, B**). In addition, the germination tubes were always visible along with secondary hyphae formation in WT at 24 hpi (100 ± 30 µm), while the C4 plants barely showed few germ tubes and secondary hyphae (30 ± 20 µm). At 48 hpi, secondary hyphae were significantly longer in WT (150 ± 60 µm) than in C4 plants (100 ± 40 µm) (**Figures 3A, C**). Overall, these results indicate that the expression of the TYLCSV-C4 product influences the first stages of *On* infection, by inhibiting conidia adhesion and disturbing/delaying not only their germination but also germ tube elongation. This indicates that the inhibition of *On* infection is affected by the host genotype and possibly relies on cellular-based processes, such as local production of ROS occurring after fungal penetration and their involvement in the plant resistance responses (Lebeda et al., 2014).

### 3.4 The plant hormone metabolism is altered in TYLCSV C4-expressing tomato plants during *On* infection

Since plant pathogen defense responses are orchestrated by a tightly organized hormonal network, we further quantified key hormones in both WT and C4-expressing tomato plants at the end of the *On* inoculation experiment (15 dpi). Firstly, we concentrated on SA, as it has a prominent role in local and systemic defense against biotrophs, and it was shown to play a key role in basal defense against PM, as well as in PM resistance mediated by some genes, such as the Arabidopsis *pmr4* and tomato *Ol-1* genes (Nishimura et al., 2003; Sunarti et al., 2022). A strong decrease in the accumulation of SA following fungal infection occurred in WT plants, differently from C4-expressing plants (**Figure 4A**). This may indicate that C4 expression helps to maintain a level of SA sufficient to counteract *On* infection. Alternatively, it is possible that *On* colonization, which is more abundant in WT plants, is associated with a pathogen-mediated suppression of the SA defense pathway. This possibility is consistent with the notion that pathogen effectors favor disease establishment by suppressing plant defense mechanisms (Jones and Dangl, 2006). In further support of this hypothesis, it was previously reported that no difference in *On* susceptibility occurred in NahG tomato plants (unable to accumulate SA) compared to WT susceptible plants (Achuo et al., 2004). However, there are disagreeing reports regarding the involvement of SA during PM infection. In fact, an SA-dependent disease resistance mechanism was described for the *pmr4* Arabidopsis plants (Nishimura et al., 2003) and for the *Ol-1* partially resistant PM tomato line, irrespective of a concomitant drought stress treatment (Sunarti et al., 2022).

Therefore, the significant change in SA regulation in WT plants and the unaltered SA content in TYLCSV C4 plants upon *On* infection warrant more extensive studies to clarify the role of SA in the tomato PM pathosystem. Moreover, the importance of such studies is also supported by recent reports describing a significant overproduction of SA in other hosts infected by PM, such as wheat (Pál et al., 2013), Arabidopsis (Wang et al., 2020), and grapevine (Yin et al., 2022).

In the case of indole-3-acetic acid (IAA), an auxin signaling molecule that promotes pathogen infection (Navarro et al., 2006; Chen et al., 2007; Bielach et al., 2017; McClerklin et al., 2018), we observed that in the absence of *On* infection, C4 plants accumulated almost 2.7 less IAA compared to WT individuals (**Figure 4B**). Upon fungal inoculation, WT plant encountered a strong reduction of IAA accumulation, while only a slight but non-significant decrease occurred in C4 plants (**Figure 4B**). In general, increased IAA level can be considered as part of a defense strategy and auxins have been reported to possess fungicidal activity (Abdel-Aty, 2010). Thus, the significant reduction of IAA in WT upon PM infection is expected to contribute to the fungal proliferation, while the lack of regulation in TYLCSV C4 plants suggests that the overexpression of this viral gene disrupts the IAA dependence of the PM infection process, in a way similar to results obtained for the grapevine-PM pathosystems (Pimentel et al., 2021).

ABA has a pivotal role in regulating abiotic (mainly drought and salt stress) responses, but recent studies indicate that this hormone also plays an important function in the response of plants to pathogen attack, implying considerable crosstalk between biotic and abiotic stresses tolerance pathways. In the course of this study, we observed that WT and C4 plants accumulate similar basal levels of ABA and that, upon *On* infection, ABA levels reach a higher, though non-significant, level in the WT group of plants compared to TYLCSV C4-expressing plants (**Figure 4C**). These results partially disagree with the report of Achuo et al. (2006) showing sensitivity of *On* infection to ABA.

### 3.5 Transcriptional changes of key plant defense genes

#### 3.5.1 Pathogenesis-related genes and the stress-responsive *POD* gene

To better evaluate the effect of TYLCSV C4 expression on infection, the expression of key defense genes, i.e., pathogenesis-related (*PR*) genes and the stress-responsive gene *POD*, was analyzed at 15 dpi. The activation of defense responses *via* the SA signaling pathway is accompanied by the expression of *PR* genes (Uknes et al., 1992; Alexander et al., 1993; Anand et al., 2008; López-Gresa et al., 2016; Li T. et al., 2019). In this work, we investigated the transcriptional response of the *PR1* gene encoding an antifungal protein (Antoniw et al., 1980; Niderman et al., 1995), the *PR2* gene encoding a β-1,3-glucanase (Antoniw et al., 1980), and the *PR5* gene encoding osmotin and thaumatin-like proteins (van Loon et al., 1994; Van Loon and Van Strien, 1999), which are considered markers for the activation of the SA defense pathway upon infection by biotrophic pathogens (Ali et al., 2018).

The transcriptional profile of the *PR1a* SA marker gene (Uknes et al., 1992; Hennig et al., 1993; Halder et al., 2019) was similar in uninfected WT and C4-expressing plants; however, upon *On* infection, significantly higher *PR1a* expression occurred in C4-expressing plants (**Figure 5**), in agreement with their higher SA content (**Figure 4**). Therefore, the increased *PR1* expression (and SA accumulation) might contribute to the PM tolerance in C4 plants, in accordance with other plant defense responses activated against fungal infections and biotrophic pathogens (Uknes et al., 1992; Ali et al., 2018; Ghozlan et al., 2020).

A similar transcriptional profile during PM infection was observed for the other defense gene *PR5*, which was strongly activated in C4 plants compared to the WT controls. Such *PR5* gene activation is expected to induce the production of osmotin, a multifunctional stress-responsive protein belonging to the PR-5 defense protein family, shown to have a strong antifungal activity (Bashir et al., 2020). Thus, our results indicate that the PR5 proteins might also contribute to confer PM tolerance in C4-expressing tomato plants, possibly being involved in favoring the protection of native proteins under stress conditions (Manghwar et al., 2018).

Conversely, upon *On* infection, the *PR2* gene, encoding enzymes of the β-1,3-endoglucanase defense response family (van Loon et al., 2006), was more upregulated in infected WT plants compared to TYLCSV C4-expressing plants. Such transcriptional regulation might indicate that β-1,3-endoglucanase defense is not directly involved in the C4-associated tolerance to PM in this host. Our results are in agreement with data reported in (Satková et al., 2017) where genes encoding PR1 proteins are more highly upregulated during *On* infection in a susceptible tomato cultivar compared to genes encoding for β-1,3-endoglucanase.

Overall, we can conclude that the TYLCSV C4 gene product acts as an activator of SA in tomato, the main phytohormone inducing local and systemic-acquired resistance against biotrophic pathogens and of the downstream defen**s**e genes *PR1* and *PR5* (An and Mou, 2011; Li N. et al., 2019). This finding points to a novel role for this multifunctional protein, which can be added to the other previously described functions, such as virus movement (Jupin et al., 1994; Rojas et al., 2001), suppression of RNA silencing (Amin et al., 2011; Luna et al., 2012; Ismayil et al., 2018; Rosas-Diaz et al., 2018), symptom induction (Rigden et al., 1994), hypersensitive response (Mei et al., 2020), hyperplasia (Jing et al., 2019), and induction of drought stress tolerance in tomato (Pagliarani et al., 2022).

The observed effective defense response against tomato PM involving the phytohormone SA and the subsequent activation of the defense genes *PR1* and *PR5* fits with previous studies regarding other biotrophic pathogens, such as *Peronospora tabacina*, *Phytophthora parasitica* var. *nicotianae*, *Puccinia striiformis* f. sp. *tritici*, and tomato spotted wilt virus (Alexander et al., 1993; Cheng et al., 2013; Padmanabhan et al., 2019).

Regarding the transcriptional regulation of the *POD* gene an expression trend analogous to the *PR1* and *PR5* genes was observed (**Figure 5D**). *POD* expression is associated with the production of peroxidase, one of the most important antioxidant enzyme, contributing to alleviate the oxidative damage induced by scavenging ROS resulting from stress conditions, such as pathogen infection, as documented for other biotrophic pathogens (Do et al., 2003; Shi et al., 2014; Li et al., 2015), such as *Phytophthora infestans* and *Xanthomonas campestris* pv. *vesicatoria*.

#### 3.5.2 Early defense genes

To further assess the impact of TYLCSV C4-expression on PM infection in tomato, we analyzed the expression of two early defense genes at 8, 24, and 48 hours after *On* inoculation. The two genes considered were (i) non-expressor of PR1 (*NPR1*) (Cao et al., 1994), encoding a signaling component that acts downstream of SA (Kunkel and Brooks, 2002) to activate defense-related genes, and (ii) the *NPR1*-paralog *NPR3*, coding for a receptor of the plant hormone SA, promoting the proteasomal degradation of NPR1 (Shi et al., 2013). During our experiments, *NPR1* gene expression initially decreased and then increased upon infection in WT and TYLCSV C4-expressing plants compared to their uninfected counterparts; however, no significant difference was observed between the two genotypes at any of the different time points (**Figure 6**). Conversely, *NPR3* expression was more upregulated in C4-expressing individuals compared to WT controls at all the time points considered, but especially at 24 and 48 hpi (**Figure 6**).

Overall, this confirms the concept that SA regulates the degradation of *NPR1* mediated by the higher expression of *NPR3*, maintaining an appropriate *NPR1* level to establish a plant immune response to pathogen attack (Fu et al., 2012; Wang and Xiang, 2020), in our case mediated by the TYLCSV C4 protein. Moreover, *NPR3* acting as a receptor in the SA signaling pathway can bind to SA, regulating the content of *NPR1*, and the subsequent SA-mediated resistance response to PM, as already reported for other biotic stresses (Wang and Xiang, 2020).

## 4 Conclusions

Several geminiviruses are documented to have the ability to trigger the complex antiviral immune response of the plant cell, involving RNAi, PTI, and ETI (Teixeira et al., 2021). Such recognition scheme induces the activation of plant defense mechanisms that can protect plants against different co-infecting pathogens or against subsequent parasite attacks (Jones and Dangl, 2006; Teixeira et al., 2021). These signaling pathways act through the activity of phytohormones such as SA, being key factors of plant defense. In this study, we demonstrated that the C4 protein of a geminivirus (TYLCSV) might act as an activator of the signaling cascade and defense mechanism in tomato against the PM infection. This protein exploits the SA signaling pathway and the activation of the *PR1* and *PR5* genes, which lie behind the plant defense against biotrophic pathogens, such as *On*. Moreover, the activation of the *POD* gene in the C4-expressing plants might be associated with alleviation of oxidative damage caused by scavenging ROS, which are produced in response to the fungal infection. Such processes led to an increased tolerance of transgenic tomato plants to PM, enabling the reduction of the DI, mainly through the inhibition of conidial adhesion and sporulation and through the activation of molecular and hormonal pathways. These findings were supported by anatomic and physiological analyses showing that C4-*On* infected plants grew better and had a higher photosynthetic activity compared to WT control plants.

The C4/AC4 proteins of geminiviruses are the less conserved proteins encoded by this group of viruses (Medina-Puche et al., 2021). These proteins display a broad diversity of functions which are deployed not only during viral infection, but also during abiotic stresses. A few motifs potentially determining its localization on organelles and structures of the cell have been identified, such as myristoylation (myr), palmitoylation (pal), and a chloroplast transit peptide (cTP) sites (Medina-Puche et al., 2021); in addition, lines of experimental evidence that the C4/AC4 from TYLCV, beet curly top virus, and East African cassava mosaic virus are localized in the plasma membrane were also produced, as reviewed by Medina-Puche et al. (2021). Whether the TYLCSV C4 protein is associated with membranous structures is presently unknown. Indeed, while the presence of a myr site was predicted in the TYLCSV-C4 protein, no pal site could be detected using the GPS Lipid—an integrated resource for lipid modification tool (http://lipid.biocuckoo.org/webserver.php (Lebeda et al., 2014). In addition, using both the Psort (https://www.genscript.com/tools/psort) and the Balanced Subcellular Localization Predictor (http://gpcr2.biocomp.unibo.it/bacello/pred.htm), a high probability of nuclear localization was also predicted for TYLCSV-C4. As a dual membrane/nuclear localization was determined for the AC4 protein of the bipartite begomovirus mungbean yellow mosaic virus (Carluccio et al., 2018), experimental proofs of the real subcellular distribution of TYLCSV-C4 would deserve further attention.

Indeed, membrane-associated protein components involved in the resistance towards PM have been recently identified, such as proteins of the soluble N-ethylmaleimide-sensitive-factor attachment protein receptor (SNARE) complex (Pál et al., 2013; Wang et al., 2020), the actin-related protein 2/3 complex (Arp2/3 complex) (Yin et al., 2022), Rho-like GTPases (ROP) (Bielach et al., 2017), and protein encoded by *Solanum habrochaites* Oidium Resistance Required-1 gene (ShORR-1) (Chen et al., 2007). Therefore, it is tempting to speculate that localization of TYLCSV C4 on the membrane together with the above-mentioned membrane-anchored resistance associated components could reinforce the defense barriers, though by still unidentified mechanisms.

Interestingly, the perception of a biotic threat at the cell surface led to a membrane-to-chloroplast transition of the TYLCV C4 protein, reducing chloroplast-associated defense mechanisms, such as SA biosynthesis and downstream processing (Medina-Puche et al., 2020), crucial elements in the antiviral defense (Bai et al., 2005). Although it is unknown if *On* infection determines membrane-to-chloroplast translocation, our results indicate that the accumulation of SA in C4 plants infected by *On* was not impaired.

Another conserved feature of the C4/AC4 proteins from different geminiviruses is related to their ability to physically interact with receptor-like kinases (RLKs), altering signal transduction involved in the PTI response (Medina-Puche et al., 2021). As the relevance of RLKs in the initial steps of infection by extracellular pathogens such as bacteria or fungi is uncontested, future studies should be devoted to identify RLKs involved in the C4-mediated tomato resistance to PM.

On a broader perspective, the finding that TYLCSV C4 acts as a novel PM defense inducer in tomato deepens our knowledge on the mechanisms adopted by this multifunctional protein in the management of both biotic and abiotic stresses and adds new layers in the definition of the resistance mechanisms towards *On*. Overall, the obtained results indicate that the anti-PM activity based on TYLCSV-C4 operating in the tomato immune response might involve the activation of a PTI response. Future investigations will be devoted to evaluate the ability of C4 to counteract other tomato pathogens, such as *Pseudomonas syringae* pv. *tomato*, considering that common resistance-inducing genes have been recently discovered (Meng et al., 2022). Further omics-based analyses on the tomato C4 overexpressing lines will help to decipher the candidate genes responsible for the priming activity of this protein against environmental stresses.

## Data availability statement

The original contributions presented in the study are included in the article/**Supplementary Material**, further inquiries can be directed to the corresponding author/s.

## Author contributions

SM, CD, and EN conceived the study and participated in its design. SM, CD, and MF performed the experiments. CD analyzed the data. SM, CD, MP, and SP validated the results. EN, CD, and SM wrote the manuscript. All authors contributed to the article and approved the submitted version.
