# Supplementary material for: Overexpression of the C4 protein of tomato yellow leaf curl Sardinia virus increases tomato resistance to powdery mildew
Source: Front Plant Sci. 2023 Mar 31;14:1163315. doi: 10.3389/fpls.2023.1163315 (PMC10102596; doi:10.3389/fpls.2023.1163315)
Supplement: Supplementary file 1 [file DataSheet_1.pdf]

Supplementary Material

Overexpression of the C4 protein of tomato yellow leaf curl Sardinia virus increases tomato resistance to powdery mildew

Chiara D'Errico[1,2], Marco Forgia[1], Marco Pisani[3], Stefano Pavan[4], Emanuela Noris[1*] and Slavica Matić[1*]

*** Correspondence:**
Slavica Matić
slavica.matic@ipsp.cnr.it
Emanuela Noris
emanuela.noris@ipsp.cnr.it

Supplementary File 1.

>ITS sequence of the MB1 isolate of *Oidium neolycopersici*

TCGTAACAAGGTTTCCGTAGGTGAACCTGCGGAAGGATCATTACAGAGCGTGAGGCTCA
GTCGTGGCGTCAGCTGCGTGCTGGGCCGACCCTCCCACCCGTGTCGATTTCTATCTTGTT
GCTTTGGCGGGCCGGGCTACGTCGTCGCTGCCCGTACGGACATGTGTCGGCCGCCCACC
GGTTTCGACTGGAGCGCGTCCGCCAAAGACCTAACCAAAACTCATGTTGTCTTTGTCGTC
TCAGCTTTATTATTGAATTGATAAAACTTTCAACAACGGATCTCTTGGCTCTGGCATCGA
TGAAGAACGCAGCGAAATGCGATAAGTAATGTGAATTGCAGAATTTAGTGAATCATCGA
ATCTTTGAACGCACATTGCGCCCCTTGGTATTCCGAGGGGCATGCCTGTTCGAGCGTCAT
AACACCCCCTCCAGCTGCCTTTGTGTGGTTGCGGTGTTGGGGCCCGTCGCGTTGCGGCAG
CTCTTAAAGATAGTGGCGGTCCTGGCGTGGGCTCTACGCGTAGTAACTTGCTTCTCGCGA
CAGAGTGACGACAGTGGCTTGCCAAAAGCCCGTTTGTTCCAGTCACATGGATCACAG

Supplementary Figure 1. Leaf morphology in C4 transgenic tomato lines (C4-151, C4-153 and C4-156) in comparison with wild type.

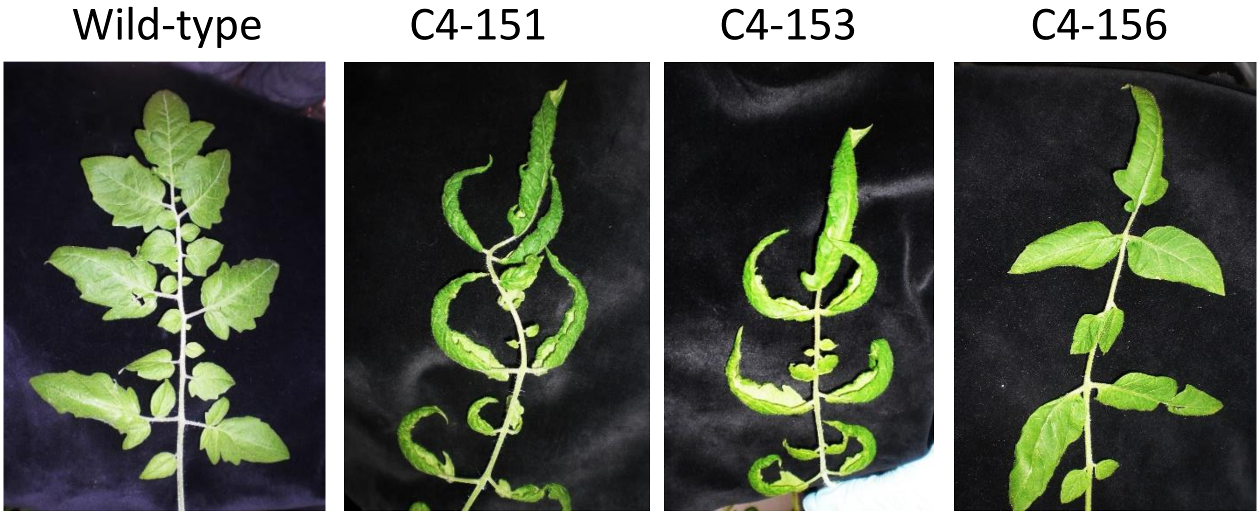

Supplementary Table 1. Impact of C4 expression on the symptom induced by *Oidium neolycopersici* on tomato

**Line**	**Percentage of infected leaves (%)**		
	**8 dpi**	**13 dpi**	**15 dpi**
C4-151	60	60	80
C4-153	60	60	80
C4-156	70	85	90
WT	100	100	100

* leaves are considered infected when fungal lesions are visually evident
